# An evaluation of the Birchwood Junk Food Café, Skelmersdale

**DOI:** 10.1093/pubmed/fdad210

**Published:** 2023-12-21

**Authors:** Greg Williams, Christine Greenhalgh, Steph Mitchell, Omer Ali, Stella Connell, David Scott, Grace Vella, Arpana Verma

**Affiliations:** Department of Public Health, Division of Population Health, Health Services Research and Primary Care, The University of Manchester, Manchester M13 9PT, UK; Department of Public Health, Division of Population Health, Health Services Research and Primary Care, The University of Manchester, Manchester M13 9PT, UK; Department of Public Health, Division of Population Health, Health Services Research and Primary Care, The University of Manchester, Manchester M13 9PT, UK; Department of Public Health, Division of Population Health, Health Services Research and Primary Care, The University of Manchester, Manchester M13 9PT, UK; The Birchwood Centre, Birch Green WN8 6QQ, UK; The Birchwood Centre, Birch Green WN8 6QQ, UK; The Birchwood Centre, Birch Green WN8 6QQ, UK; Department of Public Health, Division of Population Health, Health Services Research and Primary Care, The University of Manchester, Manchester M13 9PT, UK

**Keywords:** food choice, food waste, public health

## Abstract

Food waste is an issue of global concern requiring worldwide action. In the UK, £19 billion worth of food is wasted every year. A variety of initiatives have been developed to redistribute surplus food to those in need. The Birchwood Junk Food Café in Skelmersdale combines the reduction of food waste with community and societal benefits. The University of Manchester and the Birchwood Centre conducted an evaluation of the café including a customer satisfaction survey, a long-form health and wellbeing survey and qualitative interviews. Each day the café produces a three-course menu for the public on a ‘pay-as-you-feel’ basis. During an 18-month period, the café intercepted 32 729 kg of food that would otherwise have gone to waste, served over 1500 people, with 3500 covers, 60 different dishes and 1200 volunteer hours. Customer satisfaction was extremely high with 88% being repeated visitors and 86% rating the café as excellent. Volunteers include youth from the local Birchwood Centre, who gain valuable experiences. Customers benefit from social interactions and additional community cohesion. The café offers an unique opportunity to impact on the wider community and provides support and structure for the volunteers.

## Introduction

Food waste is an issue of global concern requiring worldwide action. In the UK, £19 billion worth of food is wasted every year.^1^ The UK government has a five-step framework known as the ‘food and drink hierarchy’, based on the EU Waste Framework Directive (2008), which prioritizes how you should deal with surplus food and drink.^2^^,^^3^ Preventing production of excess food would reduce food waste, although, most food waste occurs at household-level.^4^

### The Real Junk Food project

In response to the amount of household waste, charitable organizations have been set up to redistribute surplus food to those in need. The Real Junk Food project (RJFP) is one of those overarching groups, with a network of over 120 Junk Food cafés^5^ that take edible food from supermarkets that is often past its ‘best before’/‘display until’ date but still safe to eat and make meals for local people. Some also act as food banks to provide food for people who are unable to make ends meet. They are staffed by trained volunteers and run on a ‘pay-as-you-feel’ basis.

The Birchwood Junk Food Café in Skelmersdale combines the reduction of food waste with community and societal benefits. They developed partnerships with local supermarkets who donate food and receive donations. Each day the café is open, chefs analyse available food and produce a three-course menu for members of the public on a ‘pay-as-you-feel’ basis. Volunteer chefs are trained by senior volunteers typically working as (paid employed) chefs elsewhere. Volunteers include youth from local centres, who often go on to mentor others. Accessible pricing allows individuals on low incomes to attend and meet other people, helping reduce social isolation. Volunteers often sit with the elderly, and customers make friends at the café, building social cohesion.

## Skelmersdale

Skelmersdale sits in the borough of West Lancashire.^6^ It consists of seven wards, has higher levels of deprivation at all stages of life than elsewhere in West Lancashire and, nationally, six wards are amongst the top 20% most deprived, with four in the top 5%.^7^

The population of Skelmersdale is younger than anywhere else in West Lancashire. It has the highest proportion of children aged 0–15 living in income-deprived households and the highest proportion of adults aged 60 or over living in pension credit households.^7^

People in Skelmersdale die earlier than in other areas of West Lancashire. The lowest life expectancy at birth for males is Tanhouse ward (73.6 years), with the highest 9.4 years longer in Derby (83 years). For females, the lowest life expectancy at birth is in Birch Green (76.1 years) compared with Tarleton, 11.5 years longer (87.6 years).^7^

There are poorer levels of healthy eating and high levels of obesity among adults and children, higher levels of hospital admissions due to alcohol-related harm, higher infant mortality and higher levels of premature mortality from cancer and circulatory disease than elsewhere within West Lancashire.^7^

## BJFC

The Birchwood Centre in Skelmersdale is an independent charity that supports 13–25 year olds, providing a range of services helping young people achieve their potential. Services range from supported accommodation to intervention activities, mediation, counselling, training and development. Birchwood joined RJFP to provide healthy, cooked meals for the community.

BJFC intercepts food close to its expiry date and produces healthy meals with the help of volunteers including chefs, members of the public and young people from the Centre. For Birchwood Centre volunteers, this model provides them with the opportunity for further education and training to help enhance their employability, and interact with the community outside of the centre, enhancing life skills.

The café runs in two locations twice a week. Both centres are in deprived communities, with one located in the 3197th most deprived Lower Super Output Area (LSOA) in England and the other in the 1455th most deprived (out of 32 844 LSOAs). By locating the café in these areas, the centre helps tackle food poverty by increasing access to affordable healthy food, providing access to fresh foods and supplying hot meals to those with limited cooking facilities. It has a wide range of customers including the elderly, professionals, asylum seekers, refugees and the homeless.

The café is an asset (or safety net) within the community. Through the common activity of eating together, it provides a safe space for interaction, where the community can build friendships and networks, get to know their neighbours and feel safe. It encourages customers to learn more about food access, use and waste, and interact with more vulnerable people.

The café operates the ‘pay-as-you-feel’ system where customers decide how much to pay for their food. If customers do not have money to pay with, they may offer their services as a volunteer. This allows for inclusivity so that everybody can access the café. Food not used is allocated to the ‘pay-as-you-feel’ shop where customers can take goods home. To serve the diverse community, pay-as-you-feel instructions are provided in four languages—English, Arabic, French and Persian.

Over a period of 18 months, the Café intercepted 32 729 kg of food that would otherwise have gone to waste, served over 1500 people, with 3500 covers, 60 different dishes and 1200 volunteer hours. Processes are designed to ensure health and safety standards are upheld and a coordinator organizes the volunteers.

Over 12 months, proceeds totalled £4820.69. This ranged from £12.35 to £224.24. On average, BJFC took in £51.18 each day it ran, with a monthly average of £400.89.

## Customer satisfaction survey

The University of Manchester and the Birchwood Centre conducted a customer satisfaction survey with 134 café customers. Of those, 118 (88%) were repeat visitors, all stated they would return, and 115 (86%) rated the café as excellent.

A free text option was provided. All comments were overwhelmingly positive. Comments were coded by two separate researchers, resulting in 23 distinct codes. The 23 codes represent aspects of the café that the customers valued, for example, the ‘*quality of the food*’, the ‘*service and staff*’, ‘*company*’, the chance to make ‘*new friends*’ and ‘*healthy/fresh food*’ that helps to ‘*reduce food waste*’. Codes were grouped into themes and subthemes. Two headline themes were identified and labelled as customer and café. Respondents referred either to aspects of the café and what the café provided, or to themselves and the other customers.

### The Café

In the headline theme Café, we identified five separate subthemes; quality, venue, service/people/staff, ethos and financial support.

The quality, healthiness and freshness of the food provided by the café were commented on; ‘*Excellent food*’, ‘*great food*’, ‘*lovely fresh food*’. Customers valued facilities and atmosphere provided by the venues and the opportunity to have a space to gather; ‘*wonderful place*’, ‘*always made welcome*’, ‘*nice community feel*’. The volunteers and organizers of the café received a great deal of praise; ‘*lovely volunteers*’, ‘*good service*’, ‘*staff are all lovely and its really nice place to be*’*.*

Several customers made particular mention of how they valued the concept of reducing food waste; ‘*better to eat food before its thrown*’, ‘*It’s a fantastic idea. To think that all this food would go to waste otherwise!*’. Some were grateful for the pay-as-you-feel concept of the café and the opportunity to get food from the onsite shop; ‘*living below the line this week - £1 a day for food and drink so it really help!!*’, ‘*I am struggling to cope at the minute so coming here with access to a healthy meal and food to take away is amazing*’, ‘*I am disabled and under pressure from DWP so the cafe is a welcome respite!*’.

### The customers

The success of the café is also driven by its customers. We identified three subthemes that demonstrate the value added by those attending the café; social benefits, community and supporting a cause.

Social benefits were a strong theme throughout. Customers valued the opportunity to meet up with friends and family, make new friends or get out of the house and enjoy the company of others. This has benefits in reducing social isolation, which has been identified as a key issue in Skelmersdale. Comments included, ‘*Wonderful experience every week bringing the community together*’, ‘*The café is a good way to meet friends*’, ‘*It lovely to meet up once a week with friend I’d lost contact with*’, ‘*To get out the house*’.

A strong community spirit has developed around the café with people mentioning, the ‘*fantastic community spirit*’, how ‘*It helps bring the community together*’ and that the café has a ‘*fab community atmosphere*’.

Finally, customers of the café expressed their desire to help what they see as a good cause and support a community initiative they feel is of great value to the local area; ‘*The café is a good way to … support the community*’, describing why they came to the café people said to ‘*support worthy cause’*, ‘*To support the café*’, to ‘*support a brilliant initiative*’.

### Customer telephone interviews

Customers were also asked to consent to a telephone interview to discuss their thoughts and experiences. In total, six customers participated in an interview.

The most predominant theme to emerge was the social aspect of the café. Customers talked about how they value the company of others and the main reason for their attending the café was to socialize. They were also interested in the café, how it came about, operates, improves knowledge about food waste and changes attitudes to food and healthy eating. Customers also enjoyed feeling as though they were helping others by attending.

## Interviews with café organizers and volunteers

Interviews were conducted with organizers and volunteers. Two researchers interviewed the founder of the café, the manager and two volunteer chefs. The main themes to emerge were the benefit of the café to volunteers, the positive aspect of avoiding food waste and educating people that it is okay to buy and eat food that has passed its sell-by date, the benefit of providing healthy, hot meals and groceries on a pay-as-you-feel basis and the social benefits to volunteers and customers. The strongest theme was the potential of the café, how it can be taken forward and expanded.

### Potential

All interviewees mentioned the potential to expand and thrive. One participant highlighted that it is not marketed and if they were to advertise it could be much bigger, but they expressed concern that they may struggle to cope if that happened too quickly. It was mentioned they had worked hard to make sure the café was not viewed as a ‘*soup kitchen for poor people’* and one of their ambitions was to have a food van that could travel to community events, and other communities. One barrier to expansion highlighted was the lack of facilities, such as storage and refrigeration.

Other ideas were to hold a junk food festival, having the café run 5 days a week, going to schools, finding local areas to start growing produce, getting more supermarkets on board and getting more support from local councillors and more volunteers. The organizers were conflicted as they were keen to acquire premises, a base that the café could be run out of permanently, while at the same time, acknowledging that one of the main benefits of the café was operating within communities and being accessible to people who could not travel. All interviewees were keen to see the café eventually operate full time, evenings and weekends.

### Benefit to volunteers

Interviewees mentioned how volunteer numbers had increased, mainly from word-of-mouth and social media, and stressed the benefits to volunteers, including instilling a work ethic, letting people see what life beyond their own restricted environment is like and that they are not the only people with issues and/or facing difficulties. They believe volunteering helps build confidence and self-esteem and helps them be more resilient and improve their skill base.

Interviewees felt it was important that volunteers learned from their experience, whether about food waste, cooking, healthy eating, other cultures or life in general. The café is seen as a safe, protective space where volunteers could feel supported. Specific support is also offered if needed such as, help with applications, references and interview techniques. The café is also seen as somewhere that helps raise the expectations of volunteers and inspires them to believe they have opportunities they may otherwise feel are not available to them and that they can have fun while also working hard.

Volunteer success stories were shared, such as those that have gone on to gain employment or reconnect with estranged family. The café and volunteers also highlight the work of the Birchwood Centre.

### Food waste

Food waste and the ethos of the café were mentioned. The café contributing to diverting food that would otherwise be discarded was seen as an important and rewarding part of the work. Being unsure from 1 day to the next what food would be delivered was viewed as an exciting challenge. They were proud that they were educating customers about food waste and that many people would now be more prepared to cook and eat food they may previously have rejected. All interviewees highlighted that the café also impacted their own attitude to food and food waste.

### Healthy eating and hot meals

Interviewees mentioned that many customers indicated that they did not really eat vegetables before and that café meals had made them realize how nice vegetables can be. As the café is provided with more fruit and vegetables than meat or fish, the customers are exposed to them regularly. They also mentioned customers who attend the café because it is pay-as-you-feel and how the café acts as an important lifeline. Again, interviewees referred to the impact on their eating habits and that now they ate more healthily than before.

### Social benefits

Social benefits featured strongly. Interviewees were keen to highlight that volunteers and customers need the company and the opportunity to make friends that the café provides. One interviewee described the café as the ‘*bait-on-the-hook*’ to get people to come along, interact and feel part of the community. The impact this can have on people’s lives was mentioned repeatedly and each interviewee had a story to share illustrating success in increasing people’s self-confidence and helping them make friends. It was also pointed out that although people may come to the café to meet people, organizers then have the opportunity to discover what other support they may need and signpost them to other services.

## Customer health and wellbeing questionnaires

A long questionnaire was developed to look at themes in more detail. The questions were taken from validated questionnaires and covered demographic information, social cohesion, the environment, food consumption, food waste, wellbeing and loneliness.^8–12^

A total of 35 customers completed the questionnaires. Of these, 71% (*n* = 24) were female, 53% (*n* = 18) gave their highest level of education as secondary compared to 47% (*n* = 16) university, 66% (*n* = 23) stated that their general health level was good, 70% (*n* = 23) felt they had enough money for daily expenses and 70% (*n* = 24) were not in employment.

In terms of social cohesion, 55.9% (*n* = 27) of respondents agreed that people were willing to help their neighbours, 45.2% (*n* = 14) disagreed that people do not share the same values and 63.6% (*n* = 27) agreed that there is always someone to help you.

### Food consumption

Of customers who visit the café, 76% (*n* = 25) usually eat breakfast, 55% (*n* = 18) lunch, 91% (*n* = 30) dinner and 42% (*n* = 14) snacks. Most (80%, *n* = 27) eat a hot meal at least once a day, but 9% (*n* = 3) eat a hot meal less than three times a week. At the weekend, 91% (*n* = 31) eat a hot meal at least once a day. On average, the café’s customers eat three items of fruit and three items of vegetables a day.

When asked to provide details regarding what they had eaten on 2 specific days in the previous week when the customers did not attend the café, a wide range of responses were provided with fruit (*n* = 16), toast (*n* = 15), porridge (*n* = 12) and biscuits (*n* = 12) featuring most frequently. Although customers generally demonstrate signs of a healthy diet, 78% (*n* = 25) believe they could eat healthier.

When asked questions relating to loneliness, 35% (*n* = 9) of those with a valid score were not lonely, 46% (*n* = 12) moderately lonely, 8% (*n* = 2) severely lonely and 12% (*n* = 3) very severely lonely. All of those that reported some form of loneliness (*n* = 7) reported a household composition of sole occupancy, whereas 50% (*n* = 8) reported loneliness in a household occupancy of greater than one person. Of those who recorded a valid score on the Warwick-Edinburgh Mental Wellbeing Scale, 53% (*n* = 16) showed no signs of depression, 20% (*n* = 6) possible depression and 27% (*n* = 8) probable depression. Similarly to loneliness, 88% (7/8) of those in sole occupancy showed signs of depression, compared to 26% (5/19) of those in a household with more than one person.

## Conclusions

The BJFC provides an outlet for food that is no longer able to be sold in supermarkets, contributing to reduction of food waste. The BJFC goes beyond the main goal of the RJFP, which is to ‘stop food waste’.^13^ The café offers an unique opportunity to impact on the wider community, promotes work of the Birchwood Centre and provides additional support and structure for the young people in the centre.

The café offers volunteers from the Birchwood Centre the opportunity to gain social skills, learn more about how other people live within their community and feel a greater sense of belonging. One of the key benefits for the young people is the opportunity to learn new skills, gain experience in a working environment to become better prepared for their future, independent living and gaining employment.

Customers that took part in the evaluation see the benefit of the café to themselves and also in promoting community cohesion. Customers use the café for a variety of reasons, but the increased feeling of community spirit and togetherness is a noticeable additional benefit. Customers state that the key reason they attend is the chance to meet new people and make friends. Additionally, customers indicated reasons such as a reason to leave the house, the food and the opportunity to get a hot and healthy meal when experiencing financial hardship. The customers overwhelmingly enjoy the presence of the café in their community.

## Funding

This work was funded by N8 AgriFood. This was a practical evaluation that was conducted for operational intelligence purposes to assist the Centre.

## Data availability

Data summaries will be available upon request, for further information please contact the lead author.

## Notes

**Greg Williams**, Senior Lecturer in Public Health, University of Manchester

**Christine Greenhalgh**, Lecturer in Public Health, University of Manchester

**Steph Mitchell**, Lecturer in Public Health, University of Manchester

**Omer Ali**, Lecturer in Public Health, University of Manchester

**Stella Connell**, Chief Executive, Birchwood Centre

**David Scott**, Homeless Officer/Coordinator, Birchwood Centre

**Grace Vella**, Birchwood Centre

**Arpana Verma**, Clinical Professor of Public Health and Epidemiology, University of Manchester
